# Progress in Medicine

**Published:** 1897-07-24

**Authors:** 


### PROGRESS IN MEDICINE.

### DISEASES OF CHILDREN.

(*Continued from page* 350, *Vol. XXI.*)

**The Feeding of Infants.**—With reference to the value of the numerous patent infants' foods now so extensively used, it is important to know their composition, and how they compare with mothers' milk. With a view to determine these points, Professor R. H. Chittenden[9] has made a careful chemical analysis of some of the best known foods for infants. His method of procedure was to prepare the food for use according to the printed instructions for a child of six months, and then ascertain the proportion of albuminoids, fat, sugar, &c., in the food as it would be administered to an infant. He points out that many of the manufacturers do not pretend to imitate breast milk, but do pretend that they have improved cows' milk, or added something which makes their food more digestible and wholesome than cows' milk. Five proprietary foods are analysed, and their chemical composition is shown in tabular form as compared with breast milk. Only two of them show a specific gravity approaching the normal standard (breast milk). For nearly all the preparations it is claimed that they will curdle like mothers' milk in soft, flocculent masses, but Dr. Chittenden points out that this is procured by means of a mixture of starch and insoluble albuminoids, and that this is a very different substance from the soluble casein which yields the curd in mothers' milk. In the matter of fat and carbohydrates most of the foods differ largely both in quantity and quality from the normal standard. It would appear from Dr. Chittenden's analysis that Nature having failed in recognising the wants of an infant's stomach, the makers of infants' foods have been careful to avoid her guidance in every particular, and strike out an entirely independent course for themselves. The author does not discuss the question of the value of these foods, but concludes that "cows' milk, modified by the addition of water, cream, and peptogenic milk powder, offers a product containing to the full extent all of the proximate principles present in human breast milk, and wholly free from extraneous admixtures."

Dr. Henry Ashby[10] writes on the German methods of infant feeding as expounded in papers by Escherich and Keilman. Dr. Escherich has been using Gärtner's "Fettmilch," which corresponds generally with the "modified" or "humanised" milk employed in this country. It is prepared by diluting fresh cows' milk with an equal quantity of sterilised water, and treating the mixture in a centrifugal machine, whence the milk flows off in two streams, one from the centre, being rich in fat, and the other from the periphery, containing skimmed milk, water, and any extraneous matter which may have found entrance. The former is the special fat milk, which contains half the quantity of casein present in ordinary cows' milk, and, in addition, the fat is in natural emulsion. Milk sugar is added, and the milk, after being sterilised, is ready for use, and will be found to approach very closely in chemical composition to breast milk. Dr. Escherich's investigation was as to the growth of over three hundred infants during the first fortnight of their existence—some of them for a longer period—when fed on (1) the breast; or (2) cows' milk and water, equal parts; or (3) cows' milk and water, one part to three; or (4) fat milk. The infants were weighed daily, and the results are considered in the light of the following physiological facts: (1) That a normal infant loses weight for the first four or five days of life, and (2) that it does not reach the same weight as at birth until from the tenth to the fourteenth day. He found that the weight of children fed on the two mixtures of cows' milk and water rises only slightly after the physiological decrease ceases, in the one case only 2·8 grms. daily, and 9 grms. in the other; while the infants fed on breast milk show a gain of 32·8 grms. per diem. He considers that this difference may affect the child's prospect of life very seriously, and that some other food must be sought for which will give results more nearly approximating to those from mothers' milk. This, he believes, he has found in the "fat milk" of Gärtner. His statistics show that the average loss from the original weight was on the tenth day as follows: Children fed on mothers' milk, 23 grms.; children fed on cows' milk and water, 1:1, 195 grms.; children fed on cows' milk and water, 1:3, 176 grms.; children fed on fat milk, 66 grms. He also finds that the slightest disturbance of digestion at this early age is followed by an immediate loss of weight, and if this factor be eliminated, his results from the fat milk are still better. The daily increase in weight, after the physiological decrease ceases, gives these results, namely, 32·8 grms. for children at the breast, 23·0 grms. for the healthy fed on fat milk, 9·3 grms. for the dyspeptic fed on fat milk, and 2·8 grms. for the children fed on cows' milk. There are many other interesting points in this careful investigation bearing on the advantages of fat milk.

The results of an investigation into the constitution and use of infants' foods was reported by Dr. Aitchison Robertson[11] to the Medico-Chirurgical Society of Edinburgh. His general conclusions were as follows: Most of these foods are composed of wheaten flour, mixed with extract of malt, which is supposed to act on the flour during the process of cooking, but if the printed directions are followed the diastatic ferment is promptly destroyed, and the starch remains unconverted. Some of them contain, in place of malt, the pancreatic ferments. These do not form a healthy food, as they lead to impairment of the child's natural digestive powers. In others, the starch has already been wholly converted, and is therefore absorbable with little or no digestive action. Finally, a number remain which consist solely of flour or unchanged starchy matter, and can serve but one purpose, namely, to act as an irritant on the intestinal mucous membrane of an infant. Dr. Robertson suggests that the sale of these foods should be restricted like that of poisonous drugs, and that every purchaser should produce a medical prescription for the variety of food ordered. In the discussion which followed, Dr. Carmichael remarked that these foods did nothing but harm if administered during the normal period of lactation, and after that period they were unnecessary, as the natural farinacea—wheat, barley, or oats—cooked in the ordinary way gave better results.

**"A Rare Fatal Disease of Infancy, with Symmetrical Changes in the Macula Lutea" (Kingdon).**—Two cases of this rare affection are recorded by Dr. Carl Koller,[12] which brings the total number of published cases to nineteen. Although no definite name has yet been settled on, and no definite pathology has been ascertained, the clinical features are in all the cases singularly uniform. The patients are almost always the offspring of Jewish parents, who have not themselves manifested any special peculiarities or diseases, hereditary or acquired. The earliest symptoms to appear are those in connection with the eyes, but they are usually overlooked, and it is not until a peculiar weakness of the back manifests itself, usually from the third to the eighth month, that medical advice is sought. The infants are unable to hold the head up, the back is weak, and the muscles are flabby. A gradual but progressive degeneration sets in, both physical and mental; the patient wastes and becomes idiotic, and the illness usually terminates fatally at or about the age of two years. The eye symptoms are probably not congenital, but develop during the first weeks or months of the child's life. Perception of light fails, the pupils react sluggishly, and in some cases there is oscillatory nystagmus. The changes revealed on ophthalmoscopic investigation are definite and uniform. The yellow spot region is the site of a whitish opacity, the centre of which shows a cherry-red spot. The discs are mostly yellowish or grey, but otherwise appear normal at first; later on, atrophy develops. The affliction is a family disease, as many as four examples having been found in one family. Kingdon and Sachs have examined cases post-mortem, and found changes in the large pyramidal cells of the cerebral cortex, which they ascribe to arrested development. Kingdon also describes a descending degeneration in the cervical part of the cord. The two cases described by Dr. Koller illustrate very well the general characteristics of this interesting disease, but one of his patients had survived to the age of four years, and was alive at the time of writing, although it appeared unlikely that life would last much longer. In the second case, a sister of the first patient, the retinal changes had not appeared at the age of two months, but the signs of muscular weakness were apparent. The author points out the similarity that exists between the disease and general paralysis, general muscular debility without outspoken paralysis, physical and mental decay, and fatal issue being common to both.

**Congenital Syphilis.**—The question as to whether a healthy mother should suckle her congenitally syphilitic child is discussed in a recent article by Dr. George Ogilvie.[13] He takes exception to the view of Henoch, supported by Coutts in his Hunterian lectures, that the only limitations to be placed on suckling by mother or wet nurse should be the presence of excoriations on her nipple, and that of ulceration or fissures about the mouth of the infected infant. He points out that it has now been definitely shown that a wet nurse may be infected by a tainted child, whether the nurse be or be not its mother; and argues that, although such infection occurs but rarely, still it is not justifiable to expose a healthy woman to the risk. The limitations of Henoch as to the abrasions on the mouth of the infant or the nipples of the mother are valueless, for these are as important when small and invisible as when of large size. He, therefore, advises that the rule of practice be to forbid the suckling of a syphilitic infant unless the mother or wet nurse is already suffering from the disease.

**Infectious Vulvo-Vaginitis.**—Dr. Herman B. Sheffield[14] has had an opportunity of studying an outbreak of infectious vulvo-vaginitis in children, and has published the results of his observations. The cause of the epidemic was not definitely ascertained, but the writer is inclined to trace it to the common use of a large bath-tub by the girls, one of whom had probably been admitted with the disease. Sixty-five cases in all were observed, the ages of the patients ranging from four to fourteen years. The vaginal secretion was in every case carefully and repeatedly examined, and found to contain pus cells penetrated and surrounded by diplococci, and large and small epithelial cells covered with diplococci. These diplococci were identical with the gonococci of gonorrhœa, and therefore the nature of the disease is evident. Only thirteen of the cases presented severe symptoms, such as scalding urine, hyperæsthesia of the vaginal mucous membrane, and more or less bleeding on manipulation. The remaining fifty-two cases experienced hardly any discomfort. In none of the cases, save the complicated ones, was there a rise of temperature. Purulent ophthalmia was the most common complication, four cases in all, and peritonitis comes next in order of frequency. The prognosis is good, in the absence of complications, but the course of the disease is often prolonged, from four weeks to four months, owing partly, the writer thinks, to the hymen interfering with thorough vaginal irrigation. As regards treatment, isolation is essential for the protection of other children. Great care must be taken to prevent the occurrence of purulent ophthalmia, and this is secured by keeping the child's hands from touching the affected parts, and by bandaging the eyes at night so as to keep out any of the pus particles in the bedclothes. Peritonitis and metritis are the results of faulty douching, the vaginal secretion being carried inwards by too violent irrigation. The writer recommends that douching be carried out with the child in the Sims' position, when the parts can be more thoroughly cleansed, and that the lotion should pass from a can not more than two feet above the level of the child's body through a soft rubber catheter extending not more than one inch beyond the vaginal orifice.

[9] N.Y. Med. Jour., July 18, 1896. [10] Med. Chronicle, July, 1896. [11] Edin. Med. Jour., Sept. and Nov., 1896. [12] Med. Record, Aug. 22, 1896. [13] The Lancet, 1896, Vol. I., p. 1,791. [14] Med. Surg. Bulletin, May 30, 1896.

